# Lithopedion—a rare complication of abdominal pregnancy: a clinical case report

**DOI:** 10.1093/omcr/omaf230

**Published:** 2025-11-26

**Authors:** Avni Kryeziu, Astrit Gashi, Lavdim Ymeri, Shend Kryeziu, Fatlinda Berisha, Vernesa Kryeziu, Lorent Sijarina, Melisa Stublla, Melinda Hysenaj

**Affiliations:** Clinic of Rheumatology, University Clinical Centre of Kosovo, Hospital and University Clinical Service of Kosovo, Prishtina 10000, Kosovo; ALMA MATER EUROPA Campus College “Rezonanca”, Prishtina 10000, Kosovo; Clinic of Obstetrics and Gynecology, University Clinical Centre of Kosovo, Hospital and University Clinical Service of Kosovo, Prishtina 10000, Kosovo; ALMA MATER EUROPA Campus College “Rezonanca”, Prishtina 10000, Kosovo; Clinic of Radiology, University Clinical Centre of Kosovo, Hospital and University Clinical Service of Kosovo, Prishtina 10000, Kosovo; Medical Doctor (MD), University of Prishtina, Prishtina 10000, Kosovo; Medical Doctor (MD), University of Prishtina, Prishtina 10000, Kosovo; Medical Doctor (MD), University of Prishtina, Prishtina 10000, Kosovo; Medical Doctor (MD), University of Prishtina, Prishtina 10000, Kosovo; Medical Doctor (MD), University of Prishtina, Prishtina 10000, Kosovo; Medical Doctor (MD), University of Prishtina, Prishtina 10000, Kosovo

**Keywords:** sexual and reproductive health, radiology and medical imaging, rheumatology

## Abstract

Lithopedion (from the Greek words ‘lithos’ meaning stone and ‘paidion’ meaning child) refers to a rare medical complication in which a fetus dies during an abdominal pregnancy and, unable to be absorbed by the body, calcifies and is gradually turned into stone. This process of calcification serves as the body’s way of protecting itself from the dead tissue, preserving the fetus inside the mother’s abdomen for many years, often without any symptoms. Lithopedion cases are extremely rare, with about 330 to 340 documented cases reported throughout 400 years of medical literature. We report a rare clinical case of retained abdominal pregnancy for decades in a 70-year-old postmenopausal female, who presented with chronic back pain. Multiple fetal bones calcified in the abdomen were observed on computed tomography.

## Introduction

Abdominal pregnancy is a rare and potentially life-threatening type of ectopic pregnancy where the embryo implants and grows in the abdominal cavity, outside the uterus. Abdominal pregnancies carry a high risk of complications, including hemorrhage (severe bleeding), damage to abdominal organs, and fetal abnormalities [1]. They are extremely rare, accounting for about 1–1.5% of all ectopic pregnancies, while lithopedion accounts for 1.5–1.8% of all abdominal pregnancies [2].

It is often difficult to diagnose because symptoms can be nonspecific and may mimic other abdominal clinical conditions. Common symptoms include abdominal pain, vaginal bleeding, and gastrointestinal discomfort. Ultrasound imaging, CT and MRI can help in diagnosis [3, 4]. Management usually involves a surgical intervention to remove the pregnancy and to address any complications. In some cases, the placenta might be left in place to avoid severe bleeding if it is too closely attached to vital organs. Given the severity and complexity of abdominal pregnancy, it requires specialized medical care and close monitoring [1, 5].

Lithopedion, also known as a ‘stone baby,’ is a rare medical complication in which a fetus dies during an abdominal pregnancy and becomes calcified within the mother’s body. This usually occurs when the fetus is too large to be reabsorbed by the body and cannot be expelled. Over time, the body protects itself by encasing the dead tissue in a calcified shell, effectively turning the fetus into a ‘stone.’ This condition can go undiagnosed for many years, as the calcified mass typically does not cause symptoms and may be discovered incidentally during medical imaging for other reasons. Lithopedion cases are extremely rare, with about 330 to 340 documented cases reported throughout 400 years of medical literature [6]. We report a rare clinical case of retained lithopedion for decades in a 70-year-old postmenopausal female, who presented with chronic back pain.

## Case report

This case is a 70-year-old female patient who was examined due to low back pain. The pain spreaded below the gluteus on both sides and radiated down the legs. It was more pronounced during movements, getting up and down, and while walking up stairs. The current complaints have appeared years ago, due to which the patient has been treated from time to time with symptomatic therapy by family doctors. After taking the anamnesis and objective examination, the patient underwent laboratory tests, a simple X-ray of the lumbosacral region, and a DEXA scan.

**Figure 1:**
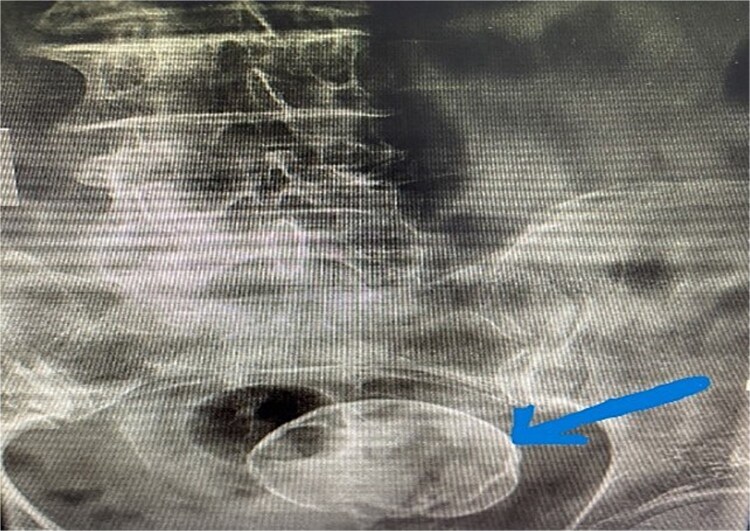
A round, radiopaque, well-circumscribed formation is seen in the pelvic region.

The laboratory tests resulted in normal values, while the x-ray of the lumbosacral region showed degenerative changes in the lumbar vertebrae, including spondylosis with osteophytes present, as well as discarthroses. At the same time, in the pelvic region, a well-defined radiopaque formation was seen, with dimensions as large as a child’s head Fig. 1.

**Figure 2:**
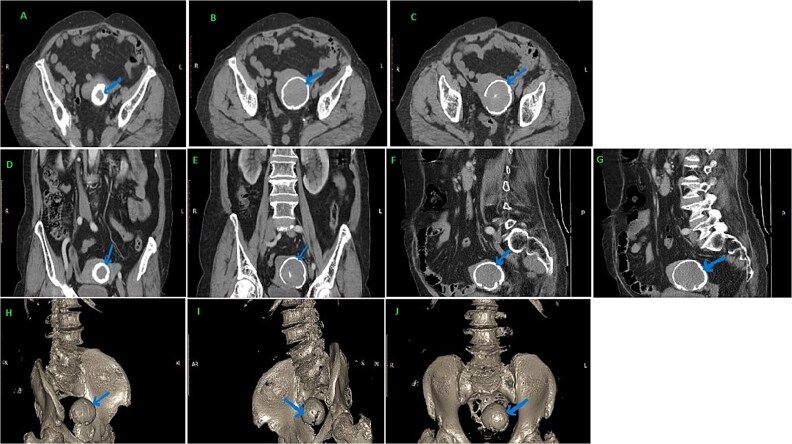
In the pelvic region, a hypodense formation is visualized, with hyperdense, thick and regular walls in the shape of a skull, with dimensions of approximately 57 × 52 mm, which initially corresponds to the head of the dead fetus, but which must be evaluated surgically (A–J).

DEXA scan showed an Osteoporosis with T-score values ​​up to −3. As soon as we saw the formation described above in the plain X-ray, the patient underwent CT of the abdominal pelvic region to clarify the findings. After performing the CT, a round formation was described, with dimensions of 59 × 52 mm, well limited with calcifications that the radiologist described as corresponding to a fetal head Fig. 2.

After this examination, a detailed obstetric and gynecological history was taken from the patient, where she emphasized that during her reproductive life, she has had no abortions and had given birth to five children. She attained menopause at 50 years of age. She said that from time to time she has had mild pain and cramping in the abdomen but that there has been no need to visit the doctor for further investigation. After such findings, we referred the patient to the gynecologist to continue treatment. The gynecologist recommended surgical intervention to remove the mass, but the patient did not consent for the same. Since there were no findings of an acute reaction and it did not present any risk to life, the patient was kept under monitoring, without proposing any alternative drug treatment. However, given the degenerative findings in the spinal column and osteoporosis, the patient was treated with anti-rheumatic therapy, mineral and vitamin supplements, chondroprotectors and bisphosphonates, according to the protocol.

## Discussion

Overall incidence of lithopedion is so low, about 0.0054% of all gestations [2]. In our country, this is the first case ever published.

Statistical data indicate that retained fetuses can persist for durations ranging from 4 to 70 years, with affected patients aged between 30 and 100 years. Kajal et al. documented a rare case involving a 60-year-old woman with a near-term fetus (37 weeks gestation) retained for 36 years [7]. Similarly, our case involves a 70-year-old patient with a retained fetus estimated to be over 20 years old.

Most lithopedion cases are asymptomatic and discovered incidentally, though some may present with chronic pelvic pain or pressure in adjacent organs [8]. In our case, diagnosis followed a rheumatologic evaluation, with the only reported symptom being lower abdominal pain noted during anamnesis.

Lithopedion can be diagnosed using abdominal X-rays, ultrasonography, and, most reliably, CT scan [9]. Imaging is essential for diagnosis, with CT scans offering greater sensitivity and specificity than X-rays. Key CT findings include a well-defined mass resembling a fetal skull, with hypodense areas and thick hyperdense calcified walls.

The standard treatment for lithopedion is elective exploratory laparotomy, allowing for intact excision of the calcified mass without intraoperative complications. A structured follow-up period is recommended to monitor for potential postoperative issues [10]. In our case, the patient declined surgical intervention for removal and histopathological confirmation via biopsy. Therefore, we recommended regular follow-up visits with a gynecologist to monitor her condition and initiate appropriate treatment if necessary.

Lithopedion, while an extraordinarily rare complication, underscores the complexities of human biology and the remarkable capacity of the body to adapt to unusual circumstances. Modern medical imaging and improved surgical techniques have greatly enhanced our ability to diagnose and manage such cases, often before complications arise. Understanding lithopedion not only enriches medical knowledge but also highlights the importance of ongoing advancements in reproductive health and prenatal care. Ultimately, each case offers unique insights into both the resilience and vulnerability of the human body.
